# Socio-economic correlates of childhood obesity in urban and rural England

**DOI:** 10.1017/S1368980023000952

**Published:** 2023-09

**Authors:** Elzbieta Titis, Jessica Di Salvatore, Rob Procter

**Affiliations:** 1 Warwick Institute for the Science of Cities, Department of Computer Science, University of Warwick, Coventry, UK; 2 Department of Politics and International Studies, University of Warwick, Coventry, UK; 3 Department of Computer Science, University of Warwick, Coventry, UK; 4 Human-Centred Computing Division, Institute for Data Science and AI, London, UK; 5 Alan Turing Institute for Data Science and AI, London, UK

**Keywords:** Supermarket proximity, Income inequality, Population density, Geography, childhood obesity, England

## Abstract

**Objective:**

Physical access to food may affect diet and thus obesity rates. We build upon existing work to better understand how socio-economic characteristics of locations are associated with childhood overweight.

**Design:**

Using cross-sectional design and publicly available data, the study specifically compares rural and urban areas, including interactions of distance from supermarkets with income and population density.

**Setting:**

We examine cross-sectional associations with obesity prevalence both in the national scale and across urban and rural areas differing in household wealth.

**Participants:**

Children in reception class (aged 4–5) from all state-maintained schools in England taking part in the National Child Measurement Programme (*n* 6772).

**Results:**

Income was the main predictor of childhood obesity (adj. R-sq=.316, p<.001), whereas distance played only a marginal role (adj. R-sq=.01, p<.001). In urban areas, distance and density correlate with obesity directly and conditionally. Urban children were slightly more obese, but the opposite was true for children in affluent areas. Association between income poverty and obesity rates was stronger in urban areas (7·59 %) than rural areas (4·95 %), the former which also showed stronger association between distance and obesity.

**Conclusions:**

Obesogenic environments present heightened risks in deprived urban and affluent rural areas. The results have potential value for policy making as for planning and targeting of services for vulnerable groups.

A growing number of studies have explored neighbourhood factors that are correlated with childhood obesity and dietary behaviours, providing conflicting evidence base, which may be due to methodological heterogeneity^(1,2)^ or complex nature of the relationship in question, meaning multiple variables must be considered^(3)^. Focussing on children is relevant for modelling future obesity rates^(4)^, as obese children tend to become obese adults^(5)^; it is also likely that prevalence of obesity in children mirrors current prevalence in adults, as obese adults tend to have obese children^(6)^. Given the likely long-term health effects children may experience due to poor nutrition^(6)^, this research responds to the call for more UK studies among children to inform public health policy and intervention design^(7)^, including rural context^(8)^.

Obesity results from positive energy imbalance and is a consequence of numerous factors, from genes, diet, levels of physical activity and the surrounding environment to social and cultural factors; e.g. it is thought to be associated with population density^(9)^, race^(10)^, unemployment level^(11)^, household income level^(12,13)^ and educational level^(9)^. Community-level environmental factors may be related with obesity rates by influencing dietary choices of individuals and promoting physical activity; for example, retail food environments (RFE) likely contribute to rising levels of obesity^(14)^, as they set the context within which people acquire food by providing opportunities and constraints that are related with food buying decisions^(15)^. Consequently, ongoing obesity crisis may be driven by qualities of the environment that promote both excess energy consumption and inadequate energy expenditure^(16)^; which may become particularly important in very restricted environments (e.g. areas of extreme poverty, rural and isolated communities, etc.)^(17)^.

A limited number of studies consider the relationship between RFE and social inequalities in diet or differences in obesity prevalence across geographical areas^(1)^. Geographical variation is important because urban and rural areas may face different barriers to eating a healthy diet, including disproportionate distribution of food sources that facilitate healthy food choices^(18)^. For example, Cummins et al.^(19)^ found associations between neighbourhood deprivation and food accessibility varied by environmental setting^(20)^; others have found that rural residents have lower food access^(21)^ or restrictive food choices^(18)^. A recent report from the UK states that about three quarters (76 %) of food deserts, FD – generally referring to low-income areas with poor geographic accessibility to a grocery store^(22)^ – in England and Wales are in urban areas, with the remaining 24 % located in relatively over-represented rural areas^(18)^. Indeed, there exists robust evidence of a positive association between living in a FD and higher childhood BMI, particularly among children in urban areas^(23)^. FD have been universally attributed to economic drivers of poor diet, such as food affordability and food prices including regional variations, but their presence remains debatable in the UK^(24)^.

This study builds upon existing work by providing a systematic analysis of which factors shape childhood BMI and under which conditions. Notably, the goal of the study is not to identify causal effects; rather, the goal is to provide a more nuanced picture of how supermarket distance and other socio-economic characteristics of locations are associated with childhood obesity. In addition to healthy food proximity and income inequality that other studies have also focussed on, this article considers associations of population density and rurality with obesity (i.e. urban *v* rural status of locations). Density and rurality have been chosen primarily because they have been shown on numerous occasions to relate to obesity in some settings but there is limited evidence for the UK. We therefore hypothesise that the relationship between supermarket distance and obesity is different in less populated areas than in more populated areas because the latter constitute an obesogenic environment. Moreover, population density is a strong predictor of proximity to the nearest supermarket^(25)^, and we would expect to see interaction between distance and density since the greater number of people living in an area should stimulate demand for food; this in turn facilitates opening of new food outlets that would translate into decreased spatial accessibility of outlets altogether, including distances to closest outlets. In addition, we also consider how rurality may interact with income in predicting obesity, as for example affluent rural areas may present less obesity-prone features compared to deprived urban areas, regardless of proximity to supermarkets.

## Data and methods

The analysis we present explores the association between children obesity rates and a set of covariates capturing physical access and economic access to food in England, using well-suited, publicly available data by the Consumer Data Research Centre (CDRC)^(26)^ and the National Child Measurement Programme (NCMP)^(27)^. The unit of analysis is the Middle Super Output Area (MSOA) level, which is a geographical unit, the NCMP data are available at translating to average populations of 7000 and an average size of 21 km^2^. Our dataset includes 6772 observations, of which 5581 and 1191 are for urban and rural areas, respectively. In the next sections, we describe the data in more detail. Geographical indicators collated at the MSOA level are widely used predictors in modelling of childhood obesity^(28,29)^ to inform interventions related to healthy nutrition practices, including preventive programmes and improving geographic access to food, as well as physical activity promotion^(30,31)^. While some research has moved to individual-level data to understand household features affecting obesity, this article focuses on how aggregated locations’ characteristics correlate with obesity rates regardless of households’ characteristic. Hence, MSOA level is suitable for investigating FD, which are indicators of food access poverty for entire areas. More importantly, aggregate-level data may provide easier recommendations for UK public health policies, which are already based on the same data.

### Dependent variable

**Fig. 1:**
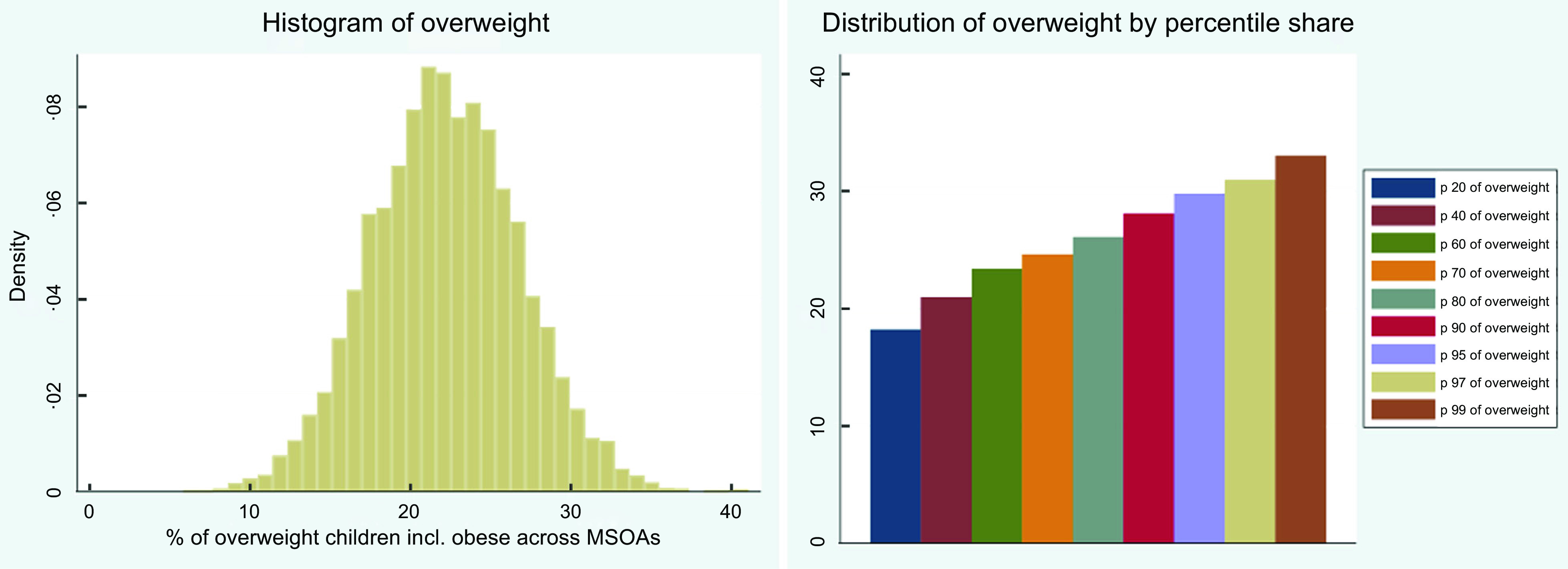
Distribution of overweight children including histogram and percentile shares

Childhood overweight data by the NCMP constituted our dependent variable measured in continuous BMI units that have been averaged across three consecutive years 2013/14, 2014/15 and 2015/16 and aggregated to 2011 MSOA level; consequently, the prevalence of overweight/obesity is in the MSOA levels rather than individual levels. Before the programme starts each school year, local authorities write to parents and carers of all children eligible for measurement to inform them of the programme. Local authorities are asked to collect data on Reception (aged 4–5) and Year 6 (aged 10–11) children’s height and weight from all state-maintained schools within their area, which then is used to produce individual-level longitudinal indicators of childhood BMI. Heights and weights are used to calculate a BMI percentile by dividing weight (in kilograms) by the square of height (in metres); children are classified as overweight (including obese) if their BMI is on or above the 85th centile of the British 1990 growth reference (UK90) according to age and sex^(27)^. The measurement process is overseen by trained healthcare professionals. Suppression and disclosure controls (numerator greater than five and a denominator of at least 50) are implemented to ensure anonymity^(27)^. Due to suppression, there was no data available for children in 19 out of 6791 MSOAs in England, giving the sample size of 6772. Figure 1 shows histogram of the variable (left panel), including distribution by percentile share (right panel).

### Independent variables

Supermarket distance, being our main independent variable, was operationalised as distance in km by car travel route from postcode centroid to the nearest outlet as calculated in 2018 by Daras et al.^(32)^. Measurement data are produced for lower super output areas, LSOA for England and Wales, and Data Zones (DZ) for Scotland. Data on roughly half a million retail businesses throughout Great Britain were provided by the Local Data Company (LDC) via the CDRC. The LDC dataset, which is regularly updated through validation via LDC field workers, includes the location of business and a hierarchical classification of the type of retail business (39 categories and 370 subcategories). The data is publicly available via CDRC^(26)^.

In addition, several socio-economic factors were collated for the purpose of this study; details on these variables and their datasets are given in Table 1, which also reports the type of measurement (e.g. distance in km), level of analysis and years covered. Finally, we also used the 2011 Rural-Urban Classification (RUC), which constituted our dummy variable for rurality. RUC is an official statistic by Office for National Statistics (ONS) used to distinguish rural and urban areas; the Classification defines areas as rural if they are outside settlements with more than 10 000 resident population. We argue that density and rurality variables measure different factors and should not be treated interchangeably. Urban units are classified as such in the UK data based on a 10 000 resident population threshold that does not capture population density and may be related to the size of the unit. Rural locations, on the other hand, have less than 10 000 inhabitants, regardless of whether the distribution is concentrated or sparse. This is further confirmed by the summary statistics showing that density in urban areas has a much higher sd than density in rural areas. This suggests an important variation in density *within* urban areas. The average density in urban areas is approximately 50, which corresponds to the maximum density recorded in rural areas.

**Table 1:** Independent variables used in the study

Variable	Full name	Dataset/Year	Level
Distance	Supermarket distance in km by car travel route from postcode centroid to nearest outlet	CDRC 2018	Postcode
Income	Average net annual household income after housing costs (£)	ONS, 2018	MSOA
Density	Population density (person/hectare)	ONS 2018	LSOA
Rurality	Urban-rural classification (0-urban, 1-rural)	ONS 2011	LSOA
% Ethnicity	Proportion of households from the ethnic minority groups to all ethnicities	Ethnic group classification, Census 2011*	LSOA
% Uneducated	Proportion of households with no qualification	Highest level of qualification, Census 2011†	LSOA
% Unemployed	Proportion of households with adults not in employment	Economic activity, Census 2011‡	LSOA

CDRC = Consumer Data Research Centre, ONS = Office for National Statistics, MSOA = middle super output area, LSOA = lower super output area.

* This dataset provides 2011 estimates that classify usual residents in England and Wales by ethnic group. The ethnic group classification used is the standard 18-category classification corresponding to the tick box response options on the census questionnaire.

† This dataset provides 2011 estimates that classify usual residents aged 16 and over in England and Wales by their highest level of qualification. This information identifies educational achievement across the population to help government resource allocation and policy making, especially in relation to disadvantaged population groups and educationally deprived areas.

‡ This dataset provides 2011 estimates that classify usual residents aged 16–74 in England and Wales by economic activity. The census concept of economic activity is compatible with the standard for economic status defined by the International Labour Organisation (ILO). It is one of a number of definitions used internationally to produce accurate and comparable statistics on employment, unemployment and economic status.

*–‡The estimates are as at census day 27 March 2011.

### Preparation and statistical description of the variables

Scatterplot matrices, Cook’s distance (any point over 4/n -where n is the total number of data points) and LOWESS (locally weighted scatterplot smoothing) tool were used to help identify outliers; the latter also allows to help diagnose non-linearities. As a result, Isles of Scilly was identified as an outlier and removed from the analysis, which resulted in 6771 observations for England and 5580 observations for urban areas; there were no outliers detected for rural areas (*n* 1191). Next, correlation matrix using Pearson’s coefficients was used to check the collinearity between independent variables (see Table 2); this was further extended by examining the variance inflation factors (VIF) for the regression models. Finally, after comparing a histogram of the sample data to a normal probability curve followed by examination of the QQ plot to test normality of variables, distance data were transformed using a log-10 transformation due to the strong positive skew in the distribution. With large enough sample sizes (> 30 or 40), however, the violation of the normality assumption should not cause major problems, meaning parametric procedures can be used even when the data are not normally distributed^(33)^.

**Table 2:** Correlation matrix between all the variables used in the study

	Obesity	Log(Dist)	Income	Density	Rurality	Ethnicity	EL	UL
Obesity (%)†	1·000							
Log(Dist)‡	−0·100*	1·000						
Income§	−0·561*	0·113*	1·000					
Density||	0·081*	−0·630*	0·052*	1·000				
Rurality¶,**	−0·078*	0·701*	0·077*	0·449*	1·000			
% Ethnicity‡‡	0·008	0·431*	0·074*	0·655*	0·288*	1·000		
% EL§§	0·565*	−0·107*	0·816*	0·072*	0·111*	−0·044*	1·000	
% UL||||	0·406*	−0·015	0·732*	0·153*	0·011	−0·184*	0·709*	1·000

UL = unemployment level, EL = education level, MSOA = middle super output area.

* *P* < 0·01.

† Proportion of overweight children (incl. obese) for MSOA 2013–16 (averaged) and collapsed to MSOA level.

‡ Road distance from postcode centroid to the nearest supermarket, the variable was log-transformed.

§ Total annual household income.

|| Number of persons per hectare.

¶ Urban–rural classification (0-urban, 1-rural).

** Point-biserial correlation between the dummies and the other continuous variables.

‡‡ Proportion of households from the ethnic minority groups.

§§ Proportion of households with no qualification.

|||| Proportion of households with adults not in employment.

**Fig. 2:**
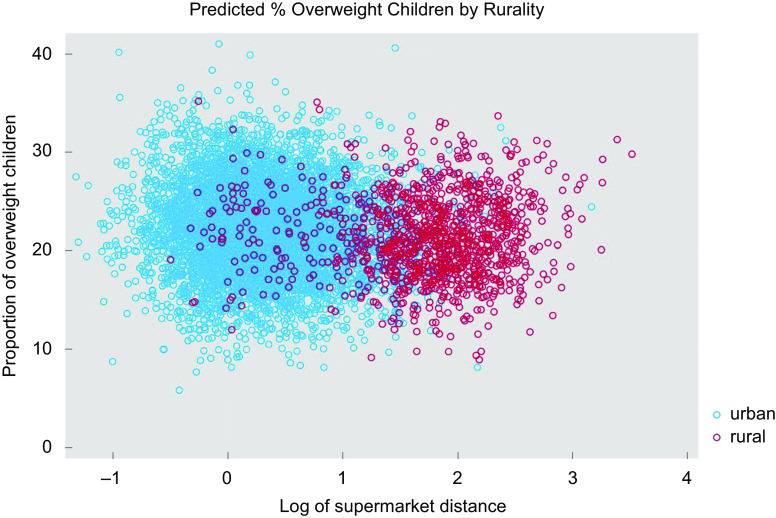
Scatterplot of overweight and log of supermarket distance by urban/rural areas

Table 3 shows the statistical descriptions for the variables used in the study. On average, nearly one-fifth (22 %) of children in the MSOA were overweight (M = 22·19, sd = 4·60), with a quarter of MSOAs (25 %) contained one in five children that were overweight. Children were more obese in urban areas (M = 22·36) when compared with rural areas (M = 21·41); unequal variance (independent) *t* test confirmed that there is a significant difference between the means of two populations (t(1877·71) = 6·9130, two-tailed *P*-value < 0·01). Percentiles for all the variables used in the analysis by urban/rural setting, including base (England), are given in online supplementary material S1. Visual examination confirmed that urban areas (*M* = 1·58) have better spatial accessibility to supermarkets than rural areas (M = 6·73) (see Fig. 2).

**Table 3:** Statistical description for the variables in the study

Variable	Mean	Std. Dev.
Childhood obesity (%)*	22·19	4·60
Log of supermarket distance†	.56	.77
Income‡	43 857·36	9720·05
Density§	41·64	36·30
% Ethnicity||	0·26	0·35
% Uneducated¶	0·23	0·08
% Unemployed**	0·33	0·07

Std. Dev. = standard deviation.

* Proportion of overweight children incl. obese, 2013–16 (averaged) and collapsed to MSOA level.

† Road distance in kilometres, the variable was log-transformed.

‡ Total annual household income in pound sterling £.

§ Number of persons per hectare.

|| Proportion of households from the ethnic minority groups.

¶ Proportion of households with no qualification.

** Proportion of households with adults not in employment.

### Modelling approach

Both main associations of income, density and rurality, and their interactions with distance were modelled using the ordinary least squares (OLS) method. Distance, income, density and rurality were used initially to set a baseline that represents the starting point of the work; once their relationship with obesity was established in terms of basic models, additional relevant covariates (i.e. ethnicity, education, employment) were included to minimise omitted-variable bias (OVB). For density and continuous income, adjusted prediction was set at various constant values with other variables set at its mean, including: (a) mean-1sd, mean and mean + 1sd; and (b) means of income quintiles. In addition, association with rurality were examined in a separate analysis, using the urban–rural dummy variable (0 for urban, 1 for rural areas) for the following: (a) below and above-average income groups, including further split by rurality, e.g. below average income in urban areas; and (b) continuous income converted into categories based on quintiles and split into rural and urban status, e.g. non-first income quintile for urban areas.

The relation for England was analysed first, followed by comparison between rural and urban areas. The data were first examined visually, and then the Likelihood Ratio Test (LRT) was used to compare the models in a systematic manner. First, the model with only distance as predictor was compared against models including one extra predictor at a time, i.e. income, density and rurality. A model that was significantly different from this initial model became a new benchmark against which models with the remaining predictors were compared one at a time. The resulting main model was then used as a final benchmark for comparisons with corresponding nested models including interaction terms. This statistical approach was appropriate as we were interested in nested model comparisons tested via chi-square difference tests comparing changes in model fit associated with the addition or removal of the parameters. After fitting the final models, regression diagnostics were used to evaluate assumptions for linear regression (see online supplementary material S2). All analyses were conducted using Stata 16.

## Results

We report similar results for England and urban areas, which is not surprising given that in 2011, 81·5 per cent (45·7 million) of the usually resident population of England and Wales lived in urban areas and 18·5 per cent (10·3 million) lived in rural areas^(34)^. We therefore jointly present results for England and urban areas, followed by discussing results for rural areas. For England and urban areas, the final model includes distance-density interaction and income, ethnicity, education and unemployment covariates; for rural areas, the final model includes distance-income interaction and ethnicity, education and unemployment covariates. Initial visual analysis showed that income-deprived areas, both urban and rural, exhibit higher rates of obesity than affluent areas, with income-deprived urban areas showing a stronger positive relation between distance and obesity than similar rural areas (see online Supplementary material S3).

### Results for England/urban areas

**Fig. 3:**
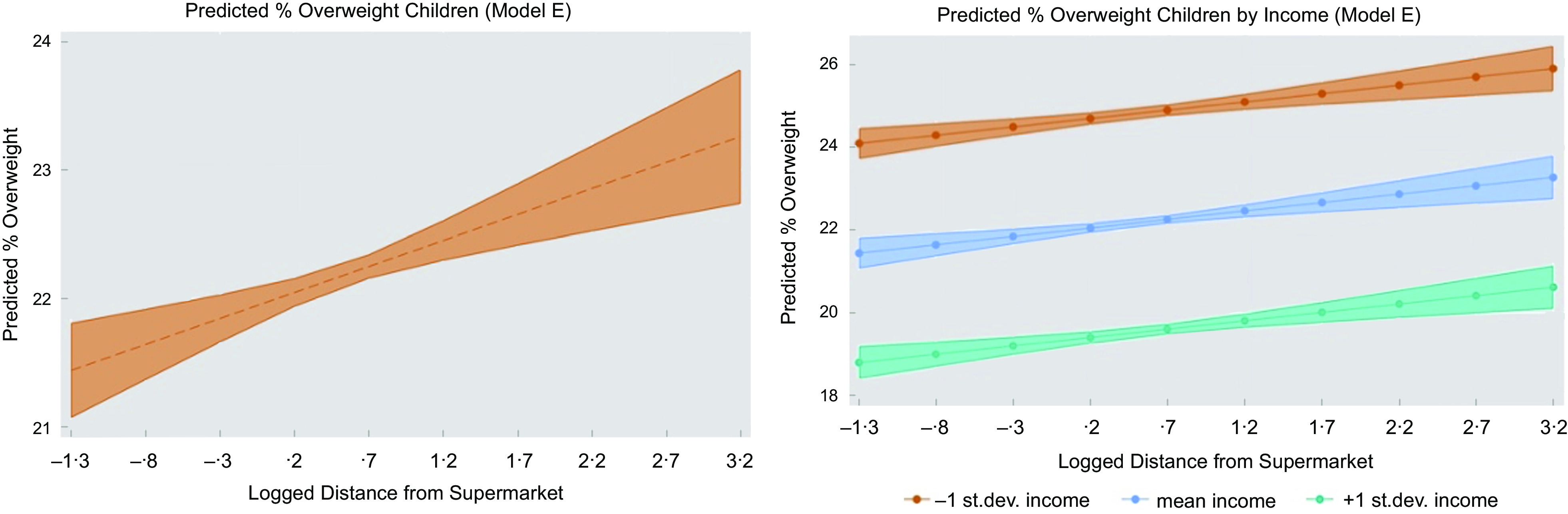
Predicted proportion of overweight children by distance and income (Model E)

Table 4 provides the summary of the OLS results for modelling main associations for England. Models A–D contain the distance predictor (our main independent variable) with one additional independent variable added one at a time; this showed that distance alone (Model A) has no large correlation (adj. Rq = 0·01, *P* < 0·001), whereas adding income to the model containing distance (Model B), helps explaining 32 % of the remaining variability in the dependent variable (adj. Rq = 0·316, *P* < 0·001). More importantly, it is only when we include population density (Model E) that distance from supermarkets reports a positive coefficient in line with existing research, suggesting that distance is associated with higher proportions of obesity among children (adj. Rq = 0·328, *P* < 0·001). This is also depicted in Fig. 3 (left panel), which shows how the proportion of overweight children increases as the distance from supermarkets grows. This seems to be true across all levels of income (Fig. 3, right panel). The LRT analysis also showed that the full specification model (Model F) including both density and rurality was not significantly different from the model including distance, income and density only (Model E). Results for urban areas are given in online Supplementary material S4. Presenting results in this consecutive manner allows for alleviating multicollinearity concerns (e.g. significance or signs flipping), as well as may indicate limitations of other studies in terms of OVB or misspecified models, hence estimating the effect of distance inaccurately. A summary of OLS results for Model E is given in Table 5 for England (column i) and urban areas (column ii); the results for fitting the interaction models are given in columns iii and column iv for England and urban areas, respectively.

**Table 4:** The associations between childhood obesity (%) and the main variables of interest (distance, income, density and rurality) in a sample of 6771 MSOA in England

	i Model A	%	ii Model B	%	iii Model C	%	iv Model D	%	v Model E	%	vi Model F	%
Dependent variable: % Childhood obesity†												
Log(Distance)‡	−0·597***	0·072	−0·224***	0·063	−0·487***	0·094	−0·534***	0·105	0·340***	0·080	0·405***	0·099
Income§	–		−0·000***	0·000	–		–		−0·000***	0·000	−0·000***	0·000
Population Density||	–		–		0·004*	0·002	–		0·019***	0·002	0·019***	0·002
Rural MSOA¶	–		–				−0·183	0·199	–		−0·190	0·170
Constant	22·526***	0·071	33·873***	0·218	22·308***	0·138	22·523***	0·071	33·162***	0·230	33·160***	0·230
Observations	6771		6771		6771		6771		6771		6771	
Adjusted *R* ^2^	0·010		0·315		0·010		0·010		0·328		0·328	

MSOA = Middle Super Output Area.

Commentary. Adding density in model E resulted in coefficient flipping for supermarket distance, which then showed a positive association with overweight in line with previous research; all the covariates are significant.

Robust standard errors in parenthesis.

* *P* < 0·10,

**P < 0·05,

*** *P* < 0·01.

† Proportion of overweight children (incl. obese), 2013–16 (averaged) and collapsed to MSOA level.

‡ Road distance from postcode centroid to the nearest supermarket, the variable was log-transformed.

§ Total annual household income.

|| Number of persons per hectare.

¶ Urban–rural classification (0-urban, 1-rural).

**Table 5:** The associations between childhood obesity (%) and other variables of interest based on model E including distance-density interaction in a sample of 6771/5580 MSOA in England/urban areas

	i England	%	ii Urban only	%	iii England	%	iv Urban only	%
Dependent variable: % Childhood obesity†								
Log(Distance)‡	0·448***	0·079	0·323**	0·115	0·461***	0·084	0·280	0·165
Population Density§	0·027***	0·002	0·026***	0·002	0·026***	0·002	0·026***	0·002
Distance × Density||	–		–		0·001	0·002	0·001**	0·003
Income¶	−0·000***	0·000	−0·000***	0·000	−0·000***	0·000	−0·000***	0·000
% Ethnicity††	−0·969***	0·191	−0·997***	0·192	−0·972***	0·192	−0·999***	0·192
% Uneducated‡‡	21·856***	1·082	22·098***	1·158	21·951***	1·101	22·038***	1·170
% Unemployed§§	−5·003***	1·044	−5·390***	1·149	−5·078***	1·048	−5·316***	1·154
Constant	24·492***	0·701	24·493***	0·744	24·483***	0·701	24·484***	0·744
Observations	6771		5580		6771		5580	
Adjusted *R* ^*2*^	0·371		0·387		0·371		0·387	
Highest VIF||||	3·53		3·61		–		–	

MSOA = middle super output area.

Robust standard errors in parenthesis.

*P < 0·10,

** *P* < 0·05,

*** *P* < 0·01.

† Proportion of overweight children (incl. obese), 2013–16 (averaged) and collapsed to MSOA level.

‡ Road distance from postcode centroid to the nearest supermarket, the variable was log-transformed.

§ Number of persons per hectare.

|| Interaction between distance and density.

¶ Total annual household income.

†† Proportion of households from the ethnic minority groups.

‡‡ Proportion of households with no qualification.

§§ Proportion of households with adults not in employment.

|||| We do not report VIF for the interaction models as adding a term that is mathematically correlated to X1 and X2 automatically increases multicollinearity.

**Fig. 4:**
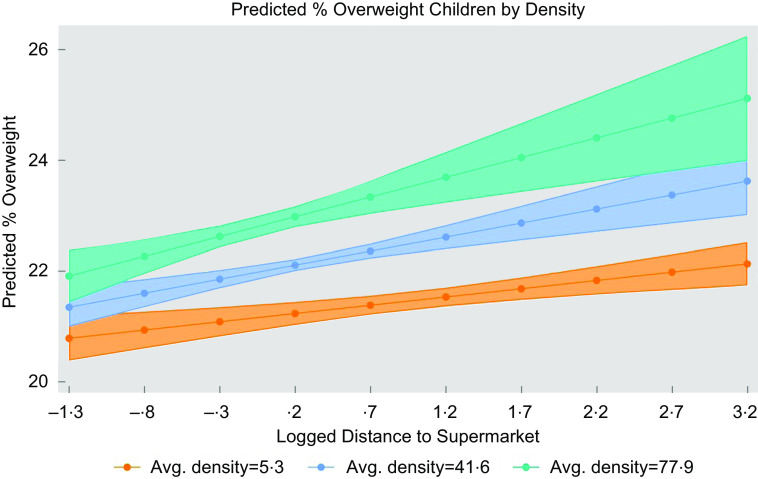
Adjusted predictions for the interaction effect distance-–density (England)

Figure 4 shows the interaction between distance and density set at low (mean –1sd), mean and high (mean + 1sd) values. The relationship between distance and obesity is stronger for densely populated areas (as given by steeper green and blue lines). The results support our hypothesis that the relationship between distance and obesity would depend on the degree of density.

### Results for rural areas

**Fig. 5:**
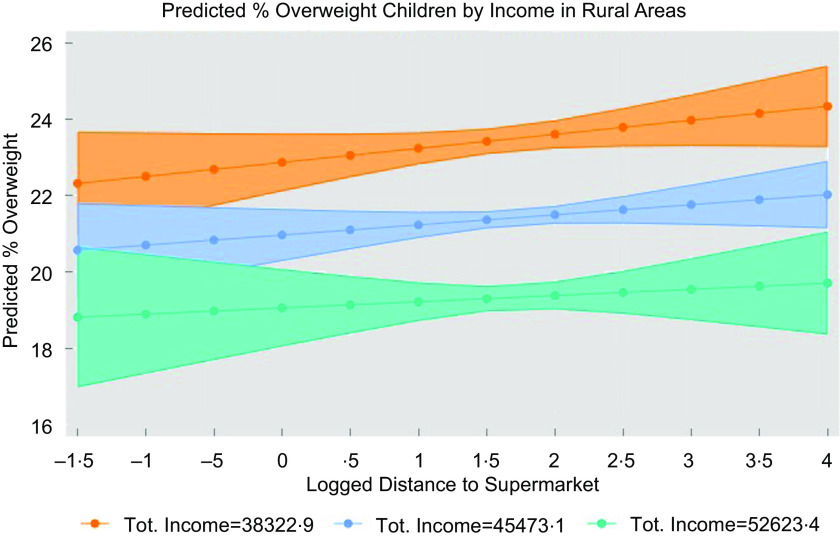
Adjusted predictions for the interaction effect distance–income (rural areas)

Table 6 provides the summary of the OLS results for modelling main associations for rural areas (column i to iv). Both income and density are significant predictors on their own, however, when both predictors are included in the model, only income remains statistically significant (*P* < 0·01, column iv). As given by the LRT analysis, modelling distance–income interaction (column vi) resulted in significantly different results from the corresponding main model including distance and income (column v). Results show that one sd increase in income (£7150·28) would result in 1·9 % percentage points decrease in overweight children. Figure 5 shows interactions for income set at low (mean –1sd), mean and high (mean + 1sd) values; the relationship between distance and obesity is slightly stronger for lower levels of income (as given by steeper blue and orange lines).

**Table 6:** The associations between childhood obesity (%) and the main variables of interest (distance, income, density), including results for the main rural model with distance-income interaction and additional covariates (Model F), in a sample of 1191 MSOA in rural areas in England

	i Model A	%	ii Model B	%	iii Model C	%	iv Model D	%	v Model E	%	vi Model F	%
Dependent variable: % Childhood obesity†												
Log(Distance)†	0·043	0·208	0·240	0·180	0·697**	0·243	0·457*	0·211	0·447*	0·183	1·364	0·984
Income§	–		−0·000***	0·000	–		0·000***	0·000	−0·000***	0·000	−0·000**	0·000
Distance × Income||	–		–		–		–		–		−0·000	0·000
Population Density¶	–		–		0·110***	0·023	0·037	0·020	–		–	
% Ethnicity††	–		–		–		–		3·656	2·937	3·107	2·974
% Uneducated‡‡	–		–		–		–		22·68***	3·431	22·816***	3·419
% Unemployed§§	–		–		–		–		−2·724	2·616	−2·705	2·613
Constant	21·340***	0·380	33·582***	0·777	19·506***	0·522	32·724***	0·877	24·375***	2·170	22·801***	2·549
Observations	1991		1991		1991		1991		1991		1991	
Adjusted *R* ^*2*^	−0·001		0·221		0·017		0·223		0·252		0·222	
Highest VIF||||	–		–		–		–		3·35		–	

MSOA = middle super output area.

Commentary. Adding density in Model D resulted in coefficient flipping for income, which then reported a positive association with overweight being contrary to previous evidence. Model F is the final model for rural areas, which includes distance–income interaction and additional socio-economic covariates (ethnicity, education, employment).

Robust standard errors in parenthesis.

* *P* < 0·10,

** *P* < 0·05,

*** *P* < 0·01.

† Proportion of overweight children (incl. obese), 2013–16 (averaged) and collapsed to MSOA level.

‡Road distance from postcode centroid to the nearest supermarket, the variable was log-transformed.

§ Total annual household income.

|| Interaction between distance and income.

¶ Number of persons per hectare.

†† Proportion of households from the ethnic minority groups.

‡‡ Proportion of households with no qualification.

§§ Proportion of households with adults not in employment.

|||| We do not report VIF for the interaction model as adding a term that is mathematically correlated to X1 and X2 automatically increases multicollinearity.

When comparing marginal effects of income set at means of income quintiles between urban and rural areas, both settings show similar rates of obesity and a clear social gradient of childhood overweight; the predicted level of overweight is 1·9 % lower when moving to a higher income quintile for urban areas, and 1·24 % lower for rural areas. Moreover, the difference in childhood overweight between 1st and 5th quintile is 7·59 % for urban and 4·95 % for rural areas; this shows that income poverty is stronger correlated with obesity rates in urban areas than in rural areas. The data are available in online supplementary material S5.

Analysis of residuals showed a minor heteroscedasticity, especially for urban areas (see online supplementary material S2), hence we used Huber-White estimator for obtaining heteroskedasticity-consistent standard errors; this should alleviate concerns of heteroscedasticity, which otherwise would violate the Gauss Markov assumptions that are necessary to render OLS the best linear unbiased estimator. Heteroscedasticity is usually mainly due to the presence of outlier in the data; however, it may also be caused due to omission of variables from the model. We therefore used the link test to examine models’ specification, being based on the idea that if a regression(-like) equation is properly specified, no additional independent variables should be significant above chance; this indeed showed that adding more variables would improve models’ estimates. It may also be that spatial variation in obesity found previously^(35)^ may affect the result, meaning using spatial models is recommended. In follow-up research, we will examine spatial dependencies and plan to include additional covariates to compare the results.

### Models with rurality variable

**Fig. 6:**
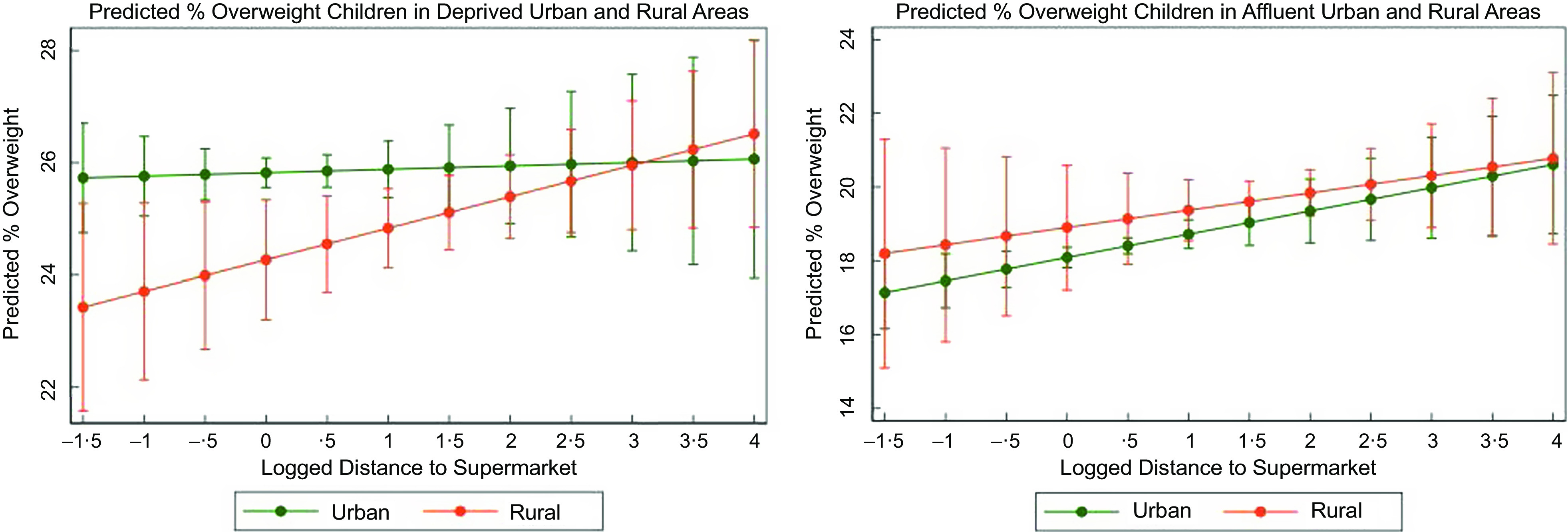
Predicted proportion of overweight children in deprived and affluent areas by rurality (urban/rural)

Additionally, a separate group of models with the rurality variable (dummy), including distance interactions with income and rurality, was used for exploratory purposes to better enunciate differences between urban and rural areas. Several models were fitted including the covariates used previously for consistency with earlier analysis. Income was measured as both continuous and dummy variables, the latter including below and above-average categories which were further split by rurality; additionally, analysis was carried out for deprived and affluent areas based on quintiles split by rurality and operationalised as first and fifth income quintiles, respectively. This analysis showed children tend to be more obese in deprived urban and affluent rural areas, but results are inconclusive (see Fig. 6); modelling predictions for deprived areas also showed that impact of distance on overweight can be conditional on lower level of income (Fig. 6, left panel). The model specifications and data are available in online supplementary material S6.

## Discussion

This study explored the relationship between supermarket proximity and obesity prevalence in school children in England, contributing to the growing UK literature on the role of RFE in influencing childhood obesity and extending existing studies by looking at the potential associations of distance with income and density both in the national scale and across urban and rural areas in England. Income has been shown to be a primary determinant of obesity, whereas distance played a marginal role; density remained important for urban areas, but not for rural areas, for which income was the only significant predictor. Locations that have both high density and are far from supermarkets had the highest levels of overweight children. Both urban and rural settings showed a positive relation between distance and obesity for different income groups, exhibiting a clear socio-economic gradient. Children in urban areas were slightly more obese, but this was not true for children in affluent areas; urban areas also showed a relatively stronger correlation between distance on obesity, and for deprived areas, this was conditional on lower level of income.

### Direct and conditional role of distance and density

We show that both density and distance had direct but also conditional associations with childhood obesity, compounding each other and being associated with higher proportion of overweight children. Moreover, population density seemed to be important only for urban areas, for which density-based measures could better capture the relation in question due to differences in service provisions (e.g. more options in urban areas including unhealthy foods which translates to shorter distances). On the other hand, proximity could be an appropriate measure for rural areas because distances from a person’s residence to nearest food outlet found there are further apart relative to distances in urban areas^(36)^, meaning ‘proximity metrics may give a reasonable measure of food access in rural areas, but they may under-estimate food access in urban areas’^(37)^. Drawing on the suggestion that different measures may be capturing different aspects of neighbourhoods^(2)^, further research is needed to compare measures in different geographical settings in the UK.

### Income had a stronger correlation with obesity in rural areas

Low income has a positive effect on childhood obesity, which is a well-known argument and thus the direct effect of income was merely our baseline hypothesis. We further show that income and education were the only two significant predictors of rural obesity, the former which may be attributed to additional geographical barriers in accessing supermarkets in rural areas^(20)^, (e.g. food requires additional economic resources for using public transport or buying fuel). Our analysis also clearly picks up a socio-economic gradient of obesity, as shown by previous research^(38)^. We show that higher income predicted smaller obesity rates in children regardless of setting, meaning that wealth is somehow universally protective against limited exposure to healthy RFE.

### Obesogenic environments present heightened risks in urban areas

Obesity rates vary considerably between urban and rural areas both in the UK and globally due to differences in geographical profiles, with the dominant paradigm attributing a higher prevalence of obesity to be persistent in urban area. A vast body of evidence relates urbanisation to rising obesity due to food options in urban areas being typically more varied and accessible than in rural areas^(39)^. On the other hand, children residing in rural households are more likely to consume healthier diets than those in urban households^(40)^ because of ‘traditional’ eating (based upon core foods such as bread, potatoes, vegetables)^(41)^, including higher fruit and vegetable intake^(42,43)^, and typically have higher levels of physical activity because of the focus on agricultural work^(44)^. Higher rates of obesity in urban areas could also be explained by the increased availability of unhealthy food options in urban areas^(45)^, transforming urban neighbourhoods into so-called food swamps. Urban dwellers may also exhibit less-traditional eating habits, such as eating away from home, that are promoted by increasing wealth^(46)^. Our results confirm that children in urban areas tended to be slightly more obese than children in rural areas; this, however, was not true for children in affluent rural areas, which tended to be more obese than their counterparts in affluent urban areas. This shows that differences between the two affluent populations mainly relate to factors associated with rurality and not income, which may be due to various barriers to healthier living, some of which are unique to rural status (e.g. lack of weight loss resources in rural communities, lack of exercise facilities and lack of low fat or low-calorie options in grocery stores)^(47)^.

### Obesity is likely dependent on the combination of deprivation and geography

Our results support previous evidence that residents of deprived neighbourhoods in rural and remote areas have poorer spatial accessibility to grocery stores^(20)^. We also show a slightly stronger association between distance and overweight in urban areas being conditional on lower level of income, which adds further weight to the suggestion that FD may exist in urban areas in the UK. On the other hand, deprived rural areas exhibited a weaker relation and thus lower obesity rates than similar urban areas; this could be due to deprived rural areas having different typologies of food retailers, hence the closest one for rural households tend to be different from the closest one for urban households. Similarly, typologies of food retailers could vary between deprived and affluent areas in both settings. Our results for rural areas remain highly unpredictable, thus requiring further examination of urban–rural conditioning and trends in the UK context.

### Strengths and limitations

In this paper, we used high-quality, openly available data. The LDC dataset is regularly validated via field work, therefore is likely more accurate compared to other administrative sources. Similarly, the NCMP programme is recognised internationally as a world-class source of public health intelligence and holds UK National Statistics status. Regardless of these advantages, we recognise that the applicability of the reported associations may be limited by the cross-sectional nature of the data and the time lag between the distance and obesity data, which, however, could not be avoided due to issues with data availability. Nevertheless, the data are well-suited for our purposes to facilitate the formulation of policies targeting public health issues based on risk factors.

We used the most recent data that was available, and supermarket distance data as of 2018 became our reference dataset to which we tried to temporarily match other variables. To alleviate concerns regarding temporal misalignment between the supermarket data and other variables, we notice that supermarkets growth has stagnated since 2016; for example, supermarket numbers growth for Sainsbury, which is the second largest supermarket in the UK, had slowed down in the previous years^(48)^. Similarly, Tesco had the biggest increase in store numbers in 2014 with 246 more stores compared to the previous financial year; in comparison, 2016–2020 saw only a minor growth in store numbers^(48)^. The urban–rural status as of 2011 is also less likely to significantly change over time.

For operationalising food access, we used data on supermarket proximity, following the gold standard in FDs research. However, exposure to unhealthy food may have a stronger relationship with obesity. Therefore, consideration for additional food outlets, including both healthy and unhealthy foods, would likely increase validity of the results. We also did not cover other factors, such as affordability or quality of healthy food items, which may explain some of our results. Moreover, future research should consider additional measures (e.g. density-based) and determinants of obesity, the latter which likely confound the results. Adding these variables to the analysis, however, is problematic due to data constraints, as data may be unavailable at geographical scales required for analysis or held by commercial organisations. Using a fixed-effects model with temporal variation could help mitigate the effect of unaccounted variables that likely confound the result. It is also possible that spatial models are better suited to study links between obesity and RFE, which we plan to examine in future research. Due to these limitations, our preliminary results need to be taken with a degree of caution.

## Conclusion

This research focussed on the rural–urban divide in the context of food access and income poverty, showing that the two settings may experience various barriers to healthy eating choices. Our results open discussion into targeting the spatial context of how food proximity and household wealth correlates with childhood obesity in England, as being an important area of food access research largely unaccounted for so far in the UK. The results have potential value for policymaking in regard to planning and targeting of services for vulnerable groups, including the populations of young children in deprived urban and affluent rural areas; such targeting is necessary because of the different ways the environmental and social determinants of health impact on these populations. In general, research around the importance of environmental interventions in ‘real-world’ settings are lacking and further work must be done to consider multiple and time-varying dimensions of food access.

## Supplementary material

For supplementary material accompanying this paper visit https://doi.org/10.1017/S1368980023000952. click here to view supplementary material

## References

[1] Titis E , Procter R & Walasek L (2021) Assessing physical access to healthy food across United Kingdom: a systematic review of measures and findings. Obes Sci Pract 8, 233–246. 10.1002/osp4.563 35388348 PMC8976549

[2] Wilkins E , Morris M , Radley D et al. (2019) Methods of measuring associations between the Retail Food Environment and weight status: importance of classifications and metrics. SSM Popul Health 8, 100404. 10.1016/j.ssmph.2019.100404 31245526 PMC6582068

[3] Schuurman N , Peters PA & Oliver LN (2009) Are obesity and physical activity clustered a spatial analysis linked to residential density. Obesity 17, 2202–2209. 10.1038/oby.2009.119 19390521

[4] Freedman DS , Kettel Khan L , Serdula MK et al. (2003) The relation of menarcheal age to obesity in childhood and adulthood: the Bogalusa heart study. BMC Pediatr 3, 1–9. https://doi.org/10.1186%2F1471-2431-3-3 12723990 10.1186/1471-2431-3-3 PMC156622

[5] Magarey AM , Daniels LA , Boulton TJ et al. (2003) Predicting obesity in early adulthood from childhood and parental obesity. Int J Obes 27, 505–513. 10.1038/sj.ijo.0802251 12664084

[6] Llewellyn A , Simmonds M , Owen CG et al. (2016) Childhood obesity as a predictor of morbidity in adulthood: a systematic review and meta-analysis. Obes Rev 17, 56–67. 10.1111/obr.12316 26440472

[7] Cetateanu A & Jones A (2016) How can GPS technology help us better understand exposure to the food environment? A systematic review. SSM Popul Health 2, 196–205. 10.1016/j.ssmph.2016.04.001 28018957 PMC5165043

[8] McGrath Davis A , James RL , Curtis MR et al. (2008) Pediatric obesity attitudes, services, and information among rural parents: a qualitative study. Obesity 16, 2133–2140. 10.1038/oby.2008.312 18551114 PMC2701507

[9] Chalkias C , Papadopoulos AG , Kalogeropoulos K et al. (2013) Geographical heterogeneity of the relationship between childhood obesity and socio-environmental status: empirical evidence from Athens, Greece. Appl Geogr 37, 34–43. 10.1016/j.apgeog.2012.10.007

[10] Williams AS , Ge B , Petroski G et al. (2018) Socioeconomic status and other factors associated with childhood obesity. J Am Board Fam Med 31, 514–521. 10.3122/jabfm.2018.04.170261 29986976 PMC9118515

[11] Oddo VM , Nicholas LH , Bleich SN et al. (2016) The impact of changing economic conditions on overweight risk among children in California from 2008 to 2012. Eur J Epidemiol 70, 874–880. 10.1136/jech-2015-207117 PMC5870869 27251405

[12] Evans GW , Jones-Rounds ML , Belojevic G et al. (2012) Family income and childhood obesity in eight European cities: the mediating roles of neighborhood characteristics and physical activity. Soc Sci Med 75, 477–481. 10.1016/j.socscimed.2012.03.037 22595070

[13] Rogers R , Eagle TF , Sheetz A et al. (2015) The relationship between childhood obesity, low socioeconomic status, and race/ethnicity: lessons from Massachusetts. Child Obes 11, 691–695. 10.1089/chi.2015.0029 26562758 PMC4939441

[14] Story M , Kaphingst KM , Robinson-O’Brien R et al. (2008) Creating healthy food and eating environments: policy and environmental approaches. Annu Rev Public Health 29, 253–272. 10.1146/annurev.publhealth.29.020907.090926 18031223

[15] Lartey A , Hemrich G & Amoroso L (2016) Influencing Food Environments for Healthy Diets. Rome: Food and Agriculture Organisation of the United Nations.

[16] Mitchell NS , Catenacci VA , Wyatt HR et al. (2011) Obesity: overview of an epidemic. Psychiatr Clin North Am 34, 717–732. 10.1016/j.psc.2011.08.005 22098799 PMC3228640

[17] Lytle LA (2009) Measuring the food environment. State of the science. Am J Prev Med 36, S134–S144. 10.1016/j.amepre.2009.01.018 19285204 PMC2716804

[18] Corfe S (2018) What are the Barriers to Eating Healthily in the UK? London: The Social Market Foundation.

[19] Cummins S , Smith DM , Taylor M et al. (2009) Variations in fresh fruit and vegetable quality by store type, urban-rural setting and neighbourhood deprivation in Scotland. Public Health Nutr 12, 2044–2050. 10.1017/s1368980009004984 19243676

[20] Smith DM , Cummins S , Taylor M et al. (2010) Neighbourhood food environment and area deprivation: spatial accessibility to grocery stores selling fresh fruit and vegetables in urban and rural settings. Int J Epidemiol 39, 277–284. 10.1093/ije/dyp221 19491142

[21] Larson NI , Story MT & Nelson MC (2009) Neighborhood environments. Disparities in access to healthy foods in the U.S. Am J Prev Med 36, 74–81. 10.1016/j.amepre.2008.09.025 18977112

[22] Wrigley N (2002) ‘Food deserts’ in British cities: policy context and research priorities. Urban Studies 39, 2029–2040. 10.1080/0042098022000011344

[23] Thomsen MR , Nayga RM , Alviola PA et al. (2016) The effect of food deserts on the Body Mass Index of elementary schoolchildren. Am J Agric Econ 98, 1–18. 10.1093/ajae/aav039

[24] MacDonald L , Ellaway A , Ball K et al. (2011) Is proximity to a food retail store associated with diet and BMI in Glasgow, Scotland? BMC Public Health 11, 1–9. 10.1186/1471-2458-11-464 21663674 PMC3128028

[25] Wilde P , Llobrera J & ver Ploeg M (2011) Population density, poverty, and food retail access in the United States: an empirical approach. Int Food Agribusiness Manag Rev 17, 171–186.

[26] Consumer Data Research Centre (2022) Local Data Company – Retail Type, Vacancy and Address Data. https://data.cdrc.ac.uk/dataset/local-data-company-retail-type-vacancy-and-address-data (accessed November 2022).

[27] NHS Digital (2021) National Child Measurement Programme. https://digital.nhs.uk/services/national-child-measurement-programme/#further-information (accessed September 2022).

[28] Wilding S , Ziauddeen N , Smith D et al. (2020) Are environmental area characteristics at birth associated with overweight and obesity in school-aged children? Findings from the SLOPE (Studying Lifecourse Obesity PrEdictors) population-based cohort in the south of England. BMC Med 18, 1–13. 10.1186/s12916-020-01513-0 32188454 PMC7081603

[29] Ziauddeen N , Wilding S , Roderick PJ et al. (2020) Predicting the risk of childhood overweight and obesity at 4–5 years using population-level pregnancy and early-life healthcare data. BMC Med 18, 1–15. 10.1186/s12916-020-01568-z 32389121 PMC7212594

[30] University College London in collaboration with Public Health England (2020) Learning from Local Authorities with Downward Trends in Childhood Obesity. PHE Publications, GW-1406. https://assets.publishing.service.gov.uk/government/uploads/system/uploads/attachment_data/file/937623/Learning_from_local_authorities_Report.pdf (accessed August 2020).

[31] Sallis A , Porter L , Tan K et al. (2019) Improving child weight management uptake through enhanced National Child Measurement Programme parental feedback letters: a randomised controlled trial. Prev Med 121, 128–135. 10.1016/j.ypmed.2019.01.023 30771362

[32] Daras K , Green MA , Davies A et al. (2019) Open data on health-related neighbourhood features in Great Britain. Sci Data 6, 107. 10.1038/s41597-019-0114-6 31263099 PMC6602943

[33] Ghasemi A & Zahediasl S (2012) Normality tests for statistical analysis: a guide for non-statisticians. Int J Endocrinol Metab 10, 486–489. 10.5812/ijem.3505 23843808 PMC3693611

[34] Office for National Statistics (2013) 2011 Census Analysis – Comparing Rural and Urban Areas of England and Wales. Office of National Statistics. https://www.basw.co.uk/system/files/resources/basw_41648-6_0.pdf (accessed August 2020).

[35] Sun Y , Hu X , Huang Y et al. (2020) Spatial patterns of childhood obesity prevalence in relation to socioeconomic factors across England. ISPRS Int J Geoinf 9, 599. 10.3390/ijgi9100599

[36] Rhone A & ver Ploeg M (2019) U.S. Shoppers’ Access to Multiple Food Stores Varies by Region. https://www.ers.usda.gov/amber-waves/2019/june/us-shoppers-access-to-multiple-food-stores-varies-by-region/ (accessed August 2020).

[37] Wilkins EL , Morris MA , Radley D et al. (2017) Using Geographic Information Systems to measure retail food environments: discussion of methodological considerations and a proposed reporting checklist (Geo-FERN). Health Place 44, 110–117. 10.1016/j.healthplace.2017.01.008 28236788

[38] Stamatakis E , Wardle J & Cole TJ (2010) Childhood obesity and overweight prevalence trends in England: evidence for growing socioeconomic disparities. Int J Obes 34, 41–47. 10.1038/ijo.2009.217 PMC3865596 19884892

[39] Bleich S , Cutler D , Murray C et al. (2008) Why is the developed world obese? Annu Rev Public Health 29, 273–295. 10.1146/annurev.publhealth.29.020907.090954 18173389

[40] Morris TT & Northstone K (2015) Rurality and dietary patterns: associations in a UK cohort study of 10-year-old children. Public Health Nutr 18, 1436–1443. 10.1017/s1368980014001864. 25192031 PMC10271822

[41] Barker ME , McClean SI , Thompson KA et al. (1990) Dietary behaviours and sociocultural demographics in Northern Ireland. Br J Nutr 64, 319–329. 10.1079/bjn19900034 2223737

[42] Levin KA , Kirby J , Currie C et al. (2012) Trends in adolescent eating behaviour: a multilevel cross-sectional study of 11–15-year-olds in Scotland, 2002–2010. J Public Health (Oxf) 2012 34, 523–531. 10.1093/pubmed/fds021 22431257

[43] Morgan K , Armstrong GK , Huppert FA et al. (2000) Healthy ageing in urban and rural Britain: a comparison of exercise and diet. Age Ageing 29, 341–348. 10.1093/ageing/29.4.341 10985444

[44] Popkin BM & Gordon-Larsen P (2004) The nutrition transition: worldwide obesity dynamics and their determinants. Int J Obes 28, S2–S9. 10.1038/sj.ijo.0802804 15543214

[45] Public Health England (2018) Fast Food Outlets: Density by Local Authority in England. Density of Fast-Food Outlets in England by Local Authority and Ward: Data Tables and Metadata. https://www.gov.uk/government/publications/fast-food-outlets-density-by-local-authority-in-england/ (accessed March 2022).

[46] Sproesser G , Ruby MB , Arbit N et al. (2019) Understanding traditional and modern eating: the TEP10 framework. BMC Public Health 19, 1–14. 10.1186/s12889-019-7844-4 31791293 PMC6889524

[47] Davis AM , James RL , Curtis MR et al. (2008) Pediatric obesity attitudes, services, and information among rural parents: a qualitative study. Obesity 16, 2133–2140. 10.1038/oby.2008.312 18551114 PMC2701507

[48] Statista (2021) Statista Dossier on Supermarkets in the United Kingdom. https://www.statista.com/study/21065/supermarkets-in-the-united-kingdom-uk-statista-dossier/ (accessed March 2022).

