# Supplementary material for: Socio-economic correlates of childhood obesity in urban and rural England
Source: Public Health Nutr. 2023 Jun 5;26(9):1815–27. doi: 10.1017/S1368980023000952 (PMC10478054; doi:10.1017/S1368980023000952)

S1 Percentiles (lower, median, higher) for all the variables used in the study by urban/rural setting incl. base (England)

	Urban & Rural setting		
	**Base**	Urban	Rural
**Childhood overweight incl. obesity**			
Base	**6,771**	5,580	1,191
Lower quintile (25%)	**19.0361**	19.2308	18.4314
Median (50%)	**22.108**	22.2973	21.1765
Higher quintile (75%)	**25.2874**	25.4973	24.3056
**Distance supermarket**			
Lower quintile (25%)	**1.00631**	.94018	4.23902
Median (50%)	**1.49955**	1.32416	6.18957
Higher quintile (75%)	**2.61086**	1.91204	8.67903
**Income**			
Lower quintile (25%)	**36,700**	35,900	40,500
Median (50%)	**42,800**	42,200	44,800
Higher quintile (75%)	**49,900**	49,900	49,800
**Density**			
Lower quintile (25%)	**18.464**	27.3006	1.344
Median (50%)	**34.676**	40.3025	4.06957
Higher quintile (75%)	**52.15**	57.2223	9.372
**Ethnicity**			
Lower quintile (25%)	**0.046357**	0.06288	0.026177
Median (50%)	**0.099142**	0.150774	0.037555
Higher quintile (75%)	**0.320708**	0.420329	0.05673

S2 Regression diagnostics

Variables/residuals were examined using both statistical tests and diagnostic plots.

1. Urban areas

1.1 Influential points

Added-variable plots showing a partial correation between independent variables and % overweight

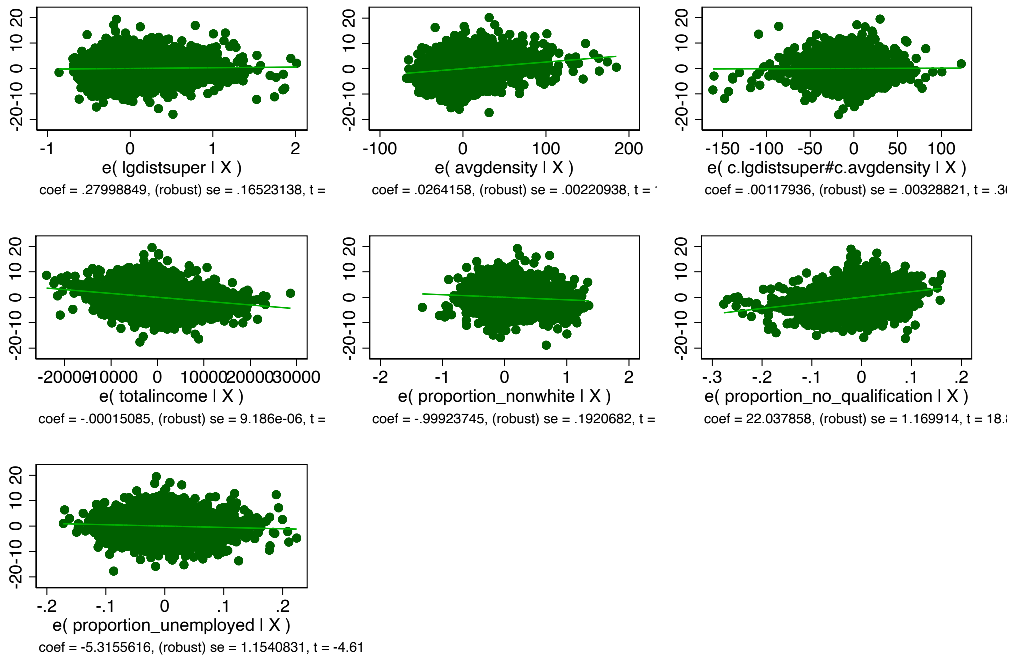

1.2 Normality of residuals

Kernel density, P-P plot and Q-Qplot for residuals

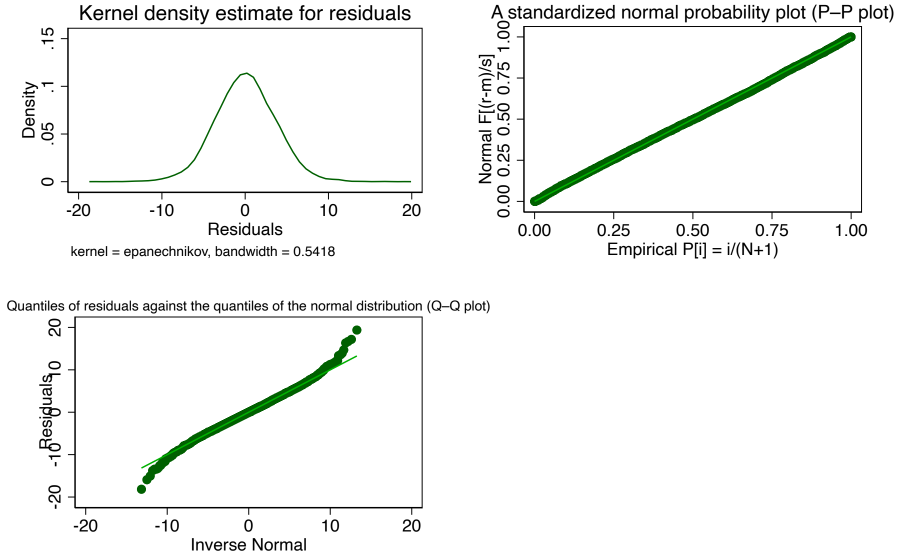

1.3 Homoscedasticity of residuals

White test: chi2(34)=246.64, Prob>chi2 = 0.0000

Breusch-Pagan test: chi2(1) = 14.31, Prob>chi2 = 0.0002

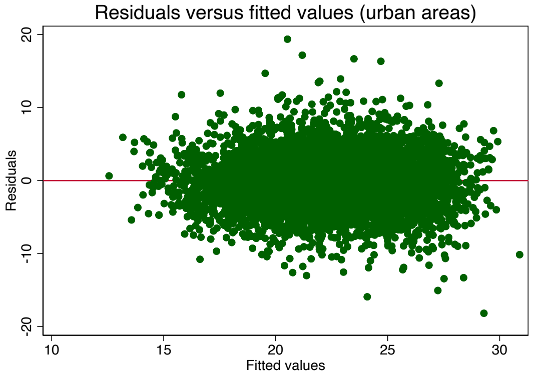

1.4 Multicollinearity

Variable	VIF (Main model)	VIF (Interaction model)
Distance	1.47	2.57
Density	2.03	2.16
Distance*Density	NA	2.59
Income	3.61	3.61
% Ethnicity	1.68	1.68
% Uneducated	3.46	3.51
% Unemployed	2.53	2.57
Mean VIF	2.46	2.67

1.5 Linearity

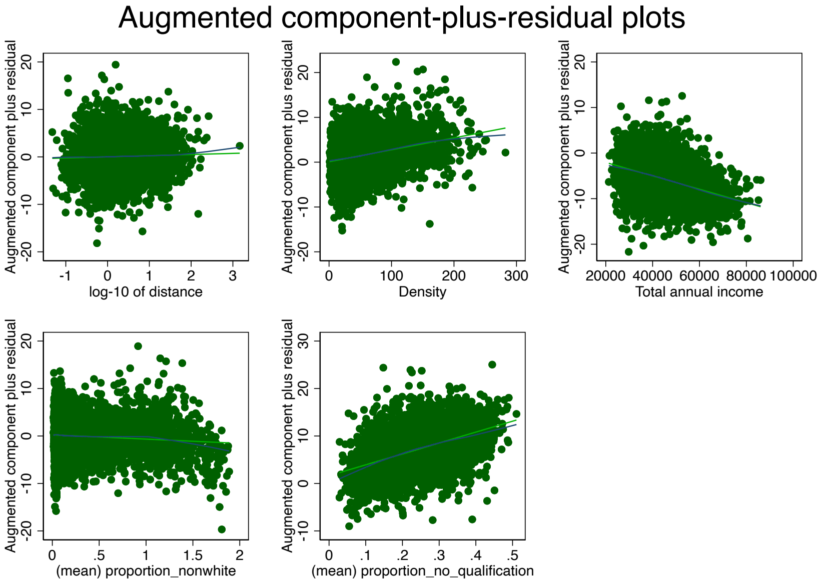

2. Rural areas

2.1 Influential points

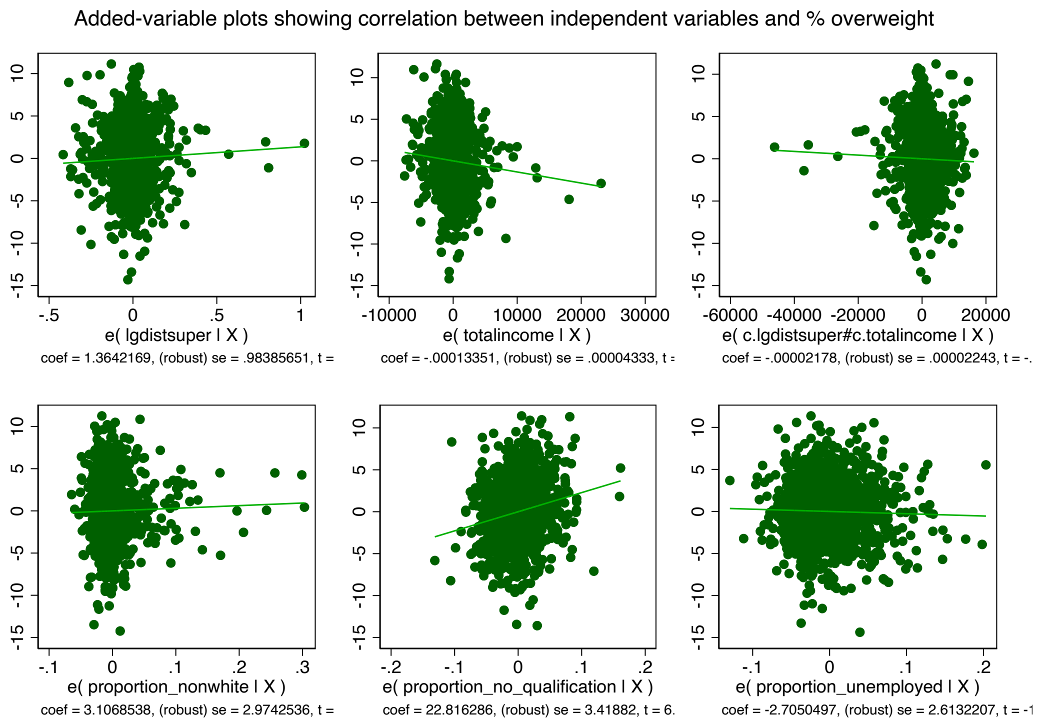

2.2 Normality of residuals

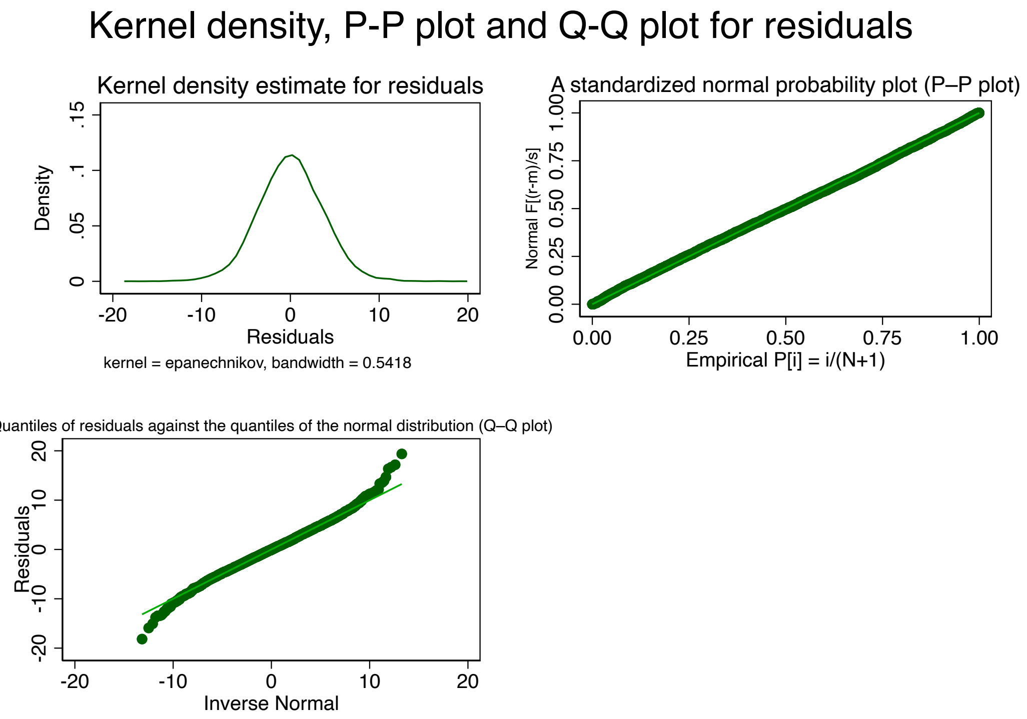

2.3 Homoscedasticity of residuals

White test: chi2(26)=32.60, Prob>chi2 = 0.1741

Breusch-Pagan test: chi2(1) = 7.29, Prob>chi2 = 0.0069

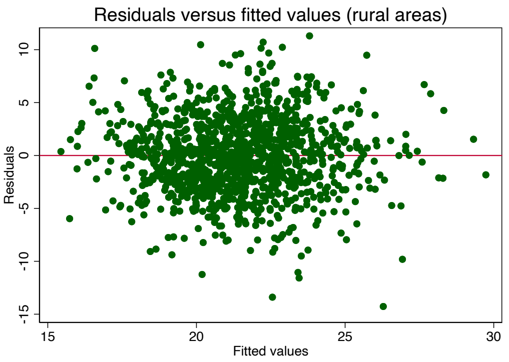

2.4 Multicollinearity

Variable	VIF (Main model)	VIF (Interaction model)*
Distance	1.10	37.34
Income	3.35	46.48
Distance*Income	NA	10.94
% Ethnicity	1.36	1.41
% Uneducated	2.67	2.68
% Unemployed	2.00	2.00
Mean VIF	2.10	16.81

* When interacting X1 and X2 (X1 * X2), we are adding a term that is mathematically correlated to X1 and X2, hence multicollinearity will increase. VIF results for the main model show no concerns of multicollinearity.

2.5 Linearity

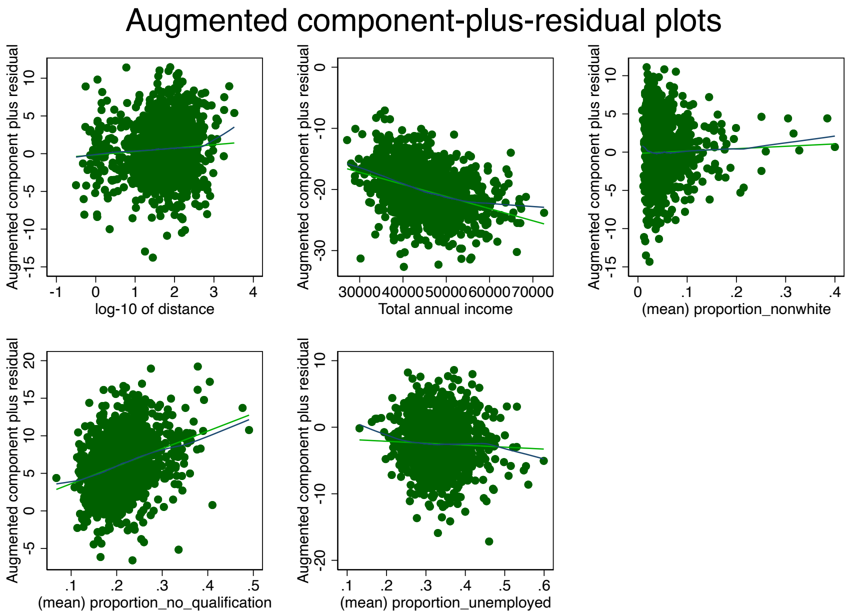

S3 Visual analysis

Scatterplots of overweight and distance by 1st and 5th income quantiles for urban vs rural areas

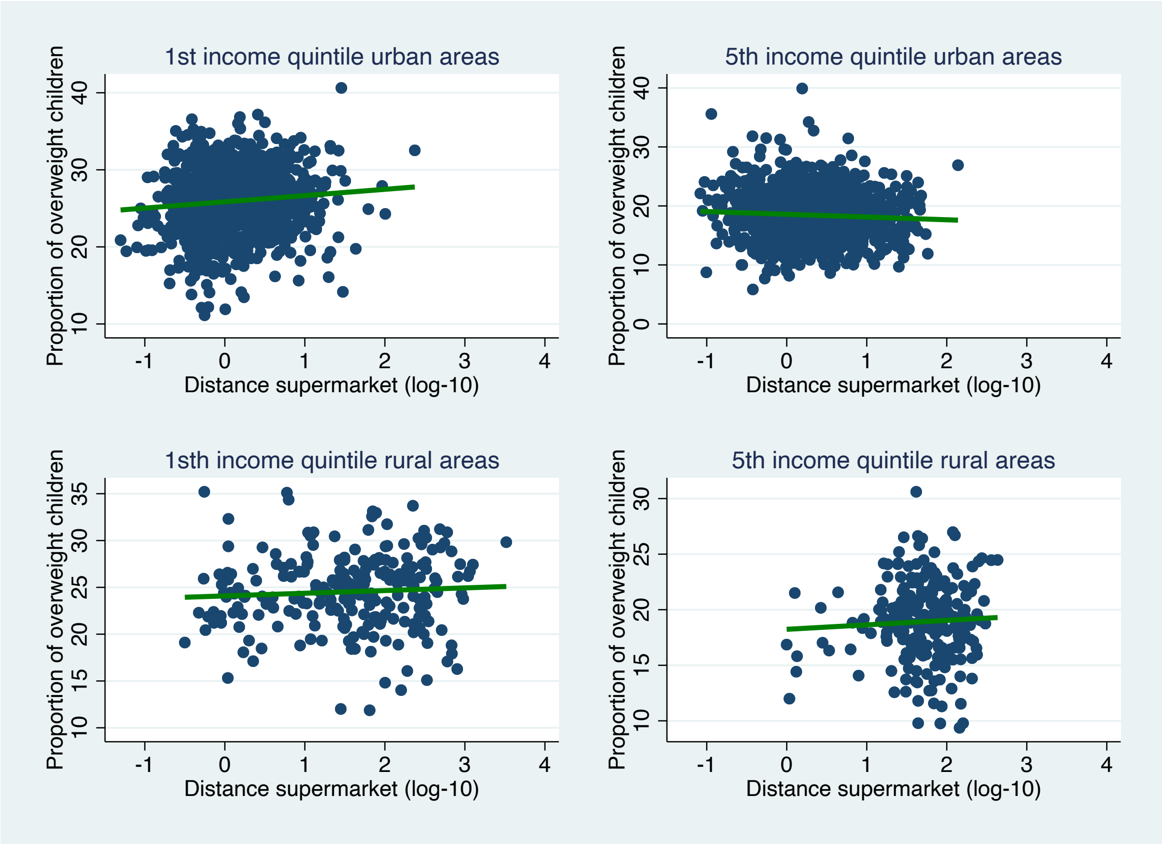

S4 The summary of the OLS results for modelling main associations for urban areas

The table below summarises the associations between childhood obesity (%) and the main variables of interest (distance, income, density), including results for the main urban model with distance-density interaction and additional covariates (Model F), in a sample of 5,580 MSOAs in urban areas in England.

	i	ii	iii	iv	v	vi
	**Model A**	**Model B**	**Model C**	**Model D**	**Model E**	**Model F**
**Dependent variable: % Childhood obesity**[1]						
Log(Distance)[2]	-0.712***	-0.654***	-0.270**	0.424***	0.323**	0.280
	(0.121)	(0.142)	(0.103)	(0.117)	(0.115)	(0.165)
Population Density[3]	-	0.002	-	0.019***	0.026***	0.026***
		(0.002)		(0.002)	(0.002)	(0.002)
Distance*Density[4]	-	-	-	-	-	0.001
						(0.003)
Income[5]	-	-	-0.000***	-0.000***	-0.000***	-0.000***
			(0.000)	(0.000)	(0.000)	(0.000)

% Ethnicity[6]	-	-	-	-	-0.997***	-0.999***
					(0.192)	(0.192)
% Uneducated[7]	-	-	-	-	22.098***	22.038***
					(1.158)	(1.170)
% Unemployed[8]	-	-	-	-	-5.370***	-5.316***
					(1.149)	(1.154)
Constant	22.578***	22.482***	33.832***	33.147***	24.493***	24.484***
	(0.074)	(0.148)	(0.229)	(0.240)	(0.744)	(0.744)
Observations	5,580	5,580	5,580	5,580	5,580	5,580
Adjusted R^2	0.006	0.006	0.328	0.341	0.387	0.387
Highest VIF[±]	-	-	-	-	3.61	-

[1] Proportion of overweight children (incl. obese), 2013-16 (averaged) and collapsed to MSOA level
[2] Road distance from postcode centroid to the nearest supermarket, the variable was log-transformed
[3] Number of persons per hectare
[4] Interaction between distance and density
[5] Total annual household income
[6] Proportion of households from the ethnic minority groups to all ethnicities
[7] Proportion of households with no qualification
[8] Proportion of households with adults not in employment
[±] We do not report VIF for the interaction model as adding a term that is mathematically correlated to X1 and X2 automatically increases multicollinearity.
* p < 0.10, ** p < 0.05, *** p < 0.01. Robust Standard Errors in parenthesis
Note. MSOA=Middle Super Output Area

S5 Marginal effects of income set at means of income quintiles: Comparison between urban and rural areas

I. Urban Areas

Margins for income quintiles and the values of distance from -1.3 to 3.5 in increments of 0.5. Based on the Model E with distance-density interaction and additional covariates given in Table 5, column iv.

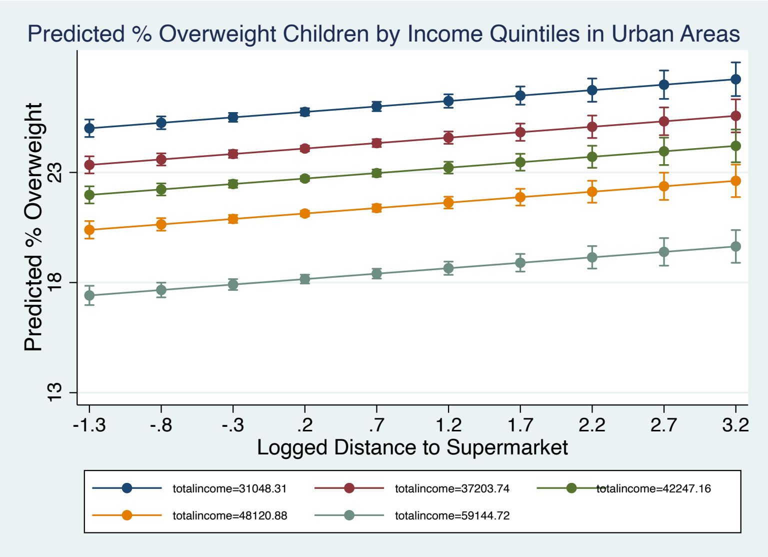

II. Rural Areas

Margins for income quintiles and the values of distance from -1.3 to 3.5 in increments of 0.5. Based on the Model F with distance-income interaction and additional covariates given in Table 6, column vi.

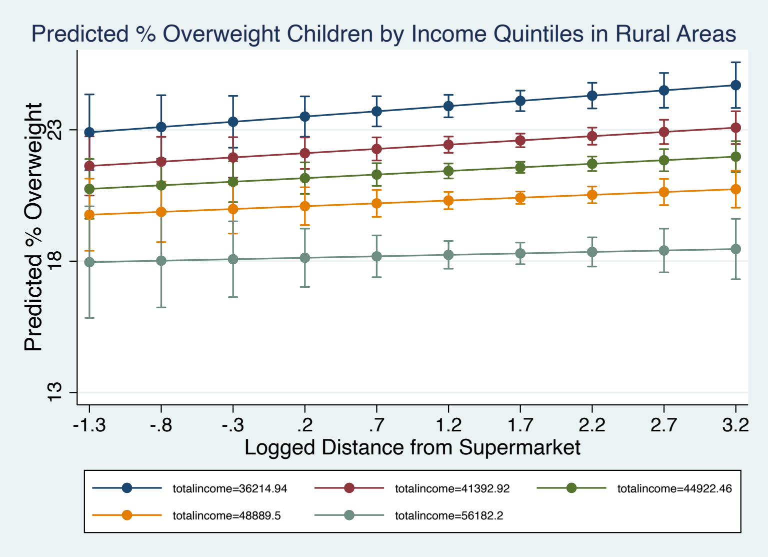

S6 Models with rurality variable

	**Model A England**	**Model B England**	**Model C England**	**Model D England**	**Model E Deprived areas±**	**Model F Affluent areas±±**
**Dependent variable: % Childhood obesity[1]**						
Distance (log-10)[2]	0.334**	0.236	0.241***	-0.008	0.0061	0.630*
	(0.115)	(0.142)	(0.124)	(0.129)	(0.283)	(0.262)
Rurality:	-0.063	-	-0.186***	-	-1.551**	0.813
0 = urban, 1= rural	(0.336)		(0.338)		(0.583)	(0.880)
Distance*Rurality	0.174	-	0.395**	-	0.501	-0.161

	(0.208)		(0.209)		(0.406)	(0.549)
Income (continuous)[3]	-0.000***	-	-	-	-	-
	(0.000)					
Income (dummy): 0 = below average, 1 = above average	-	-	-1.215***	-	-	-
			(0.155)			
Income (dummy)*Distance	-	-	-0.071		-	-
			(0.128)			
Income (below/above average) by rurality:	-		-	-	-	-
Below average, Rural		-0.224				
		(0.426)				
Above average, Urban		-1.220***				
		(0.159)				
		-1.324*				
Above average, Rural		(0.534)				
Income (below/above average) by rurality*Distance:	-		-	-	-	-
Below average, Rural		0.420				
		(0.270)				
Above average, Urban		-0.059				
		(0.206)				
		0.287				
Above average, Rural		(0.319)				
Income 1st quintile						
Non-1st rural	-	-	-	-0.755	-	-
				(0.431)		
1st urban	-	-	-	0.121	-	-
				(0.180)		
1st rural	-	-	-	0.373	-	-
				(0.529)		
1st quintile*Distance	-	-	-	0.679**	-	-
Non-1st rural				(0.257)		
	-	-	-	0.612*	-	-
1st urban				(0.289)		
	-	-	-	0.773*	-	-
1st rural				(0.773)		
Density[4]	0.026***	0.027***	0.273***	0.268***	0.017*	0.024***
	(0.002)	(0.002)	(0.002)	(0.002)	(0.007)	(0.004)
% Ethnicity[5]	-0.988***	-1.008***	-1.008***	-1.199***	-3.215***	0.885*
	(0.190)	(0.193)	(0.193)	(0.189)	(0.308)	(0.438)
% Uneducated[6]	22.015***	29.304***	29.305***	32.430***	14.490***	42.134***
	(1.090)	(0.999)	(0.994)	(0.982)	(2.016)	(2.956)
% Unemployed[7]	-5.036***	-0.591	-0.590	0.823	4.583	-9.411***
	(1.046)	(1.029)	(1.025)	(1.049)	(2.545)	(2.183)
Constant	24.434***	15.166***	15.165***	13.553***	19.401***	13.056***
	(0.231)	(0.344)	(0.344)	(0.310)	(1.001)	(0.652)
Observations	6,771	6,771	6,771	6,771	1,363	1,345
Adjusted R^2	0.371	0.351	0.352	0.344	0.142	0.187

[1] Proportion of overweight children (incl. obese), 2013-16 (averaged) and collapsed to MSOA level

[2] Road distance from postcode centroid to the nearest supermarket, the variable was log-transformed
[3] Total annual household income
[4] Number of persons per hectare
[5] Proportion of households from the ethnic minority groups to all ethnicities
[6] Proportion of households with no qualification
[7] Proportion of households with adults not in employment
* $p < 0.10$, ** $p < 0.05$, *** $p < 0.01$. Robust Standard Errors in parenthesis
±As given by the first income quintile
±±As given by the fifth income quintile

Predictive margins of rurality with 95% CIs based on Model A

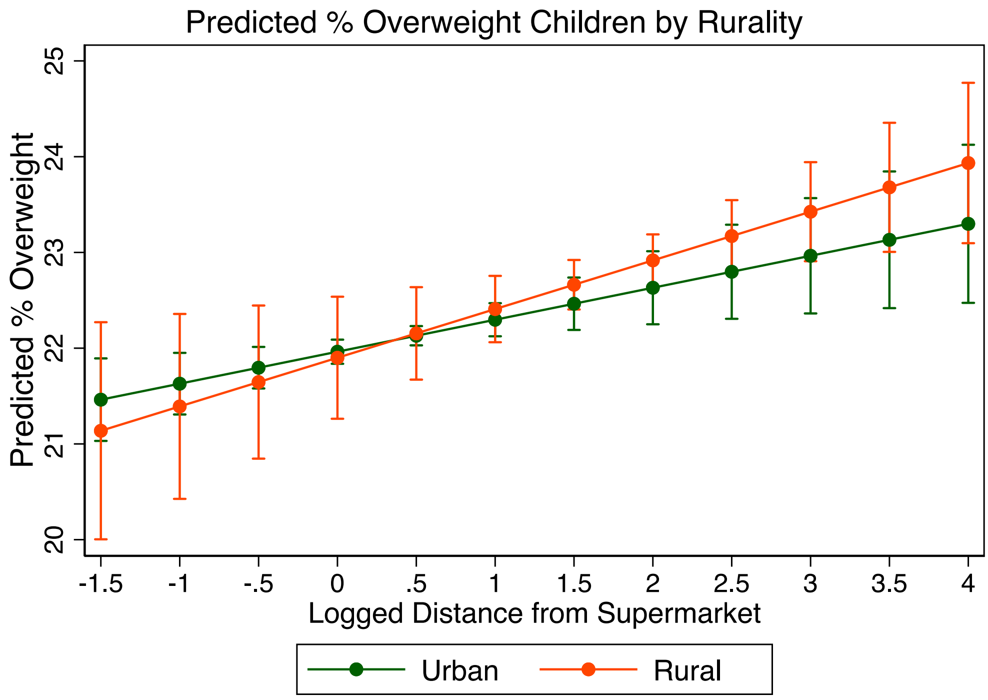

Predictive margins of income (below/above average, dummy) by rurality with 95% CIs based on Model B

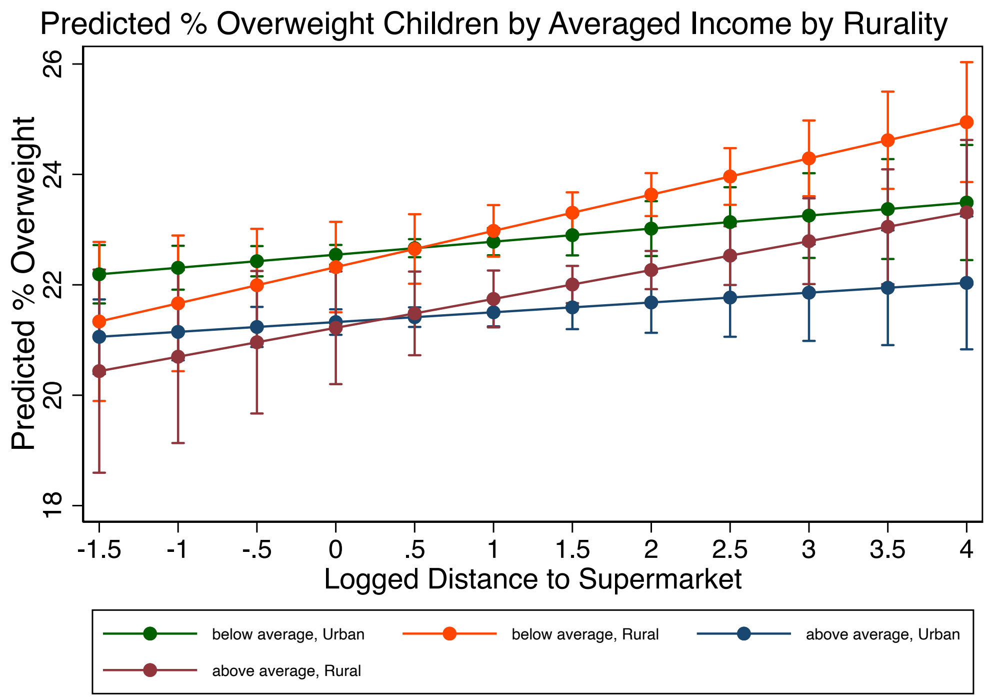

Predictive margins of income (below/above average, dummy) in urban-rural setting based on Model C

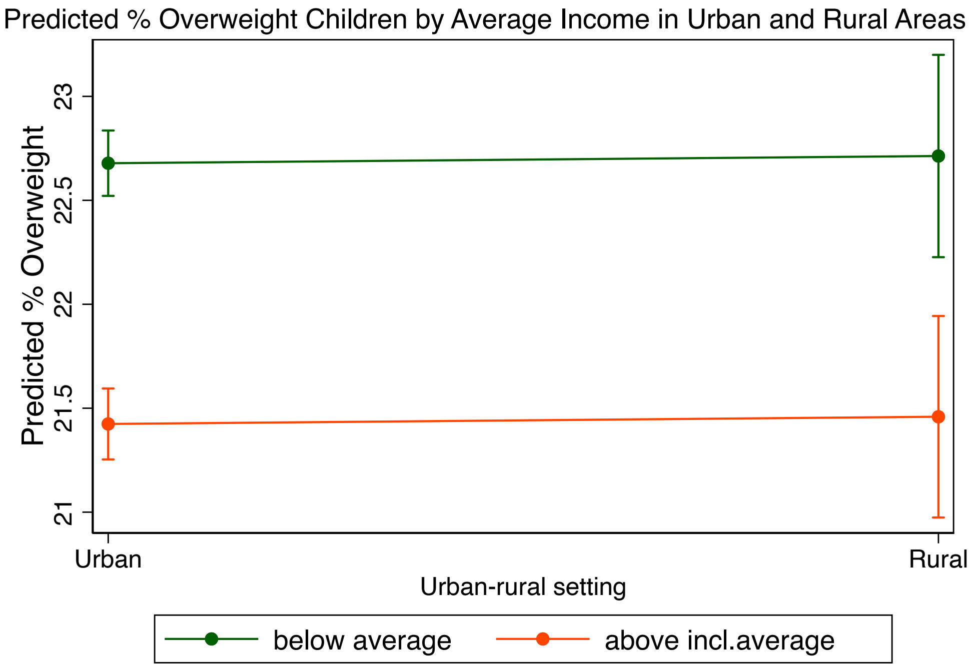

Predictive margins of income quintile (dummy) by rurality with 95% CIs based on Model D

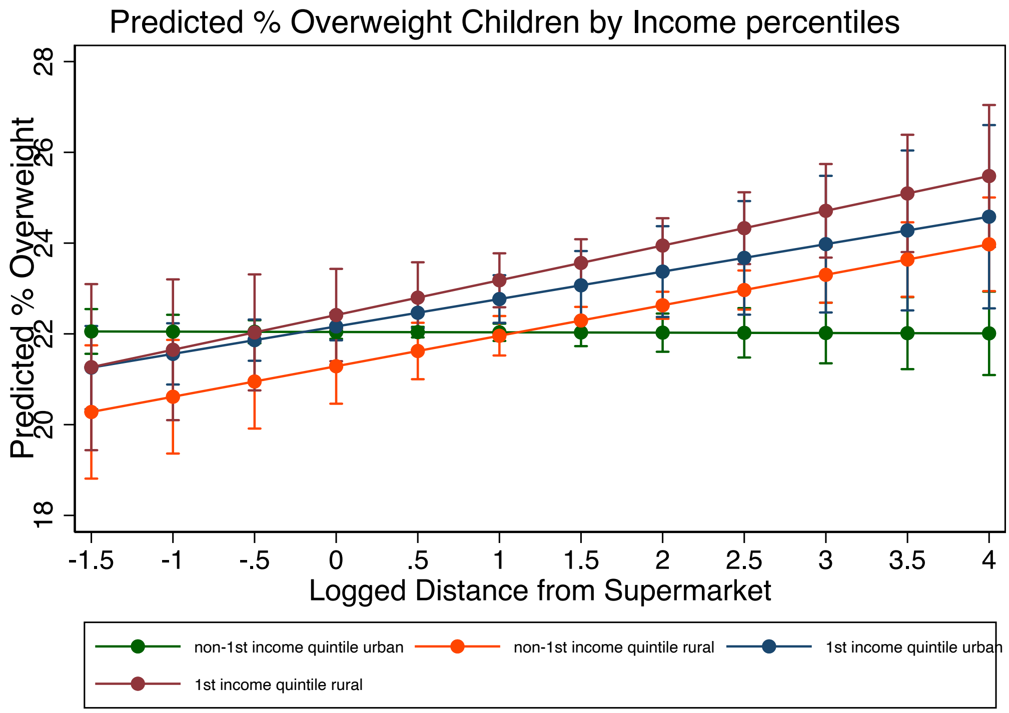

Predictive margins of rurality for income deprived areas based on Model E

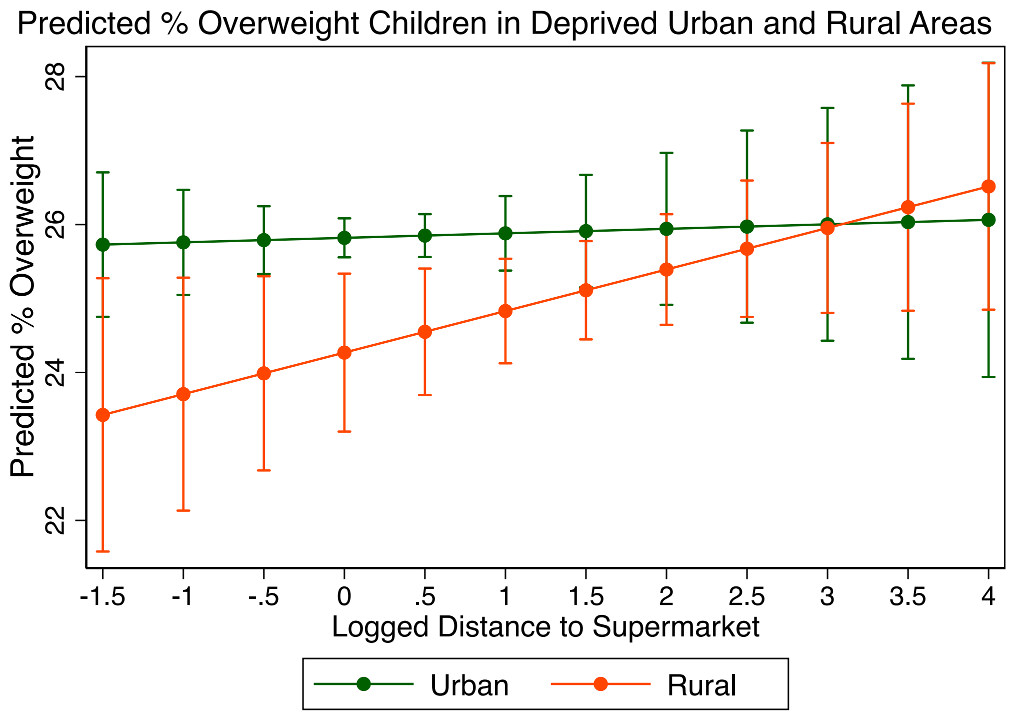

Predictive margins of rurality for income affluent areas based on Model F

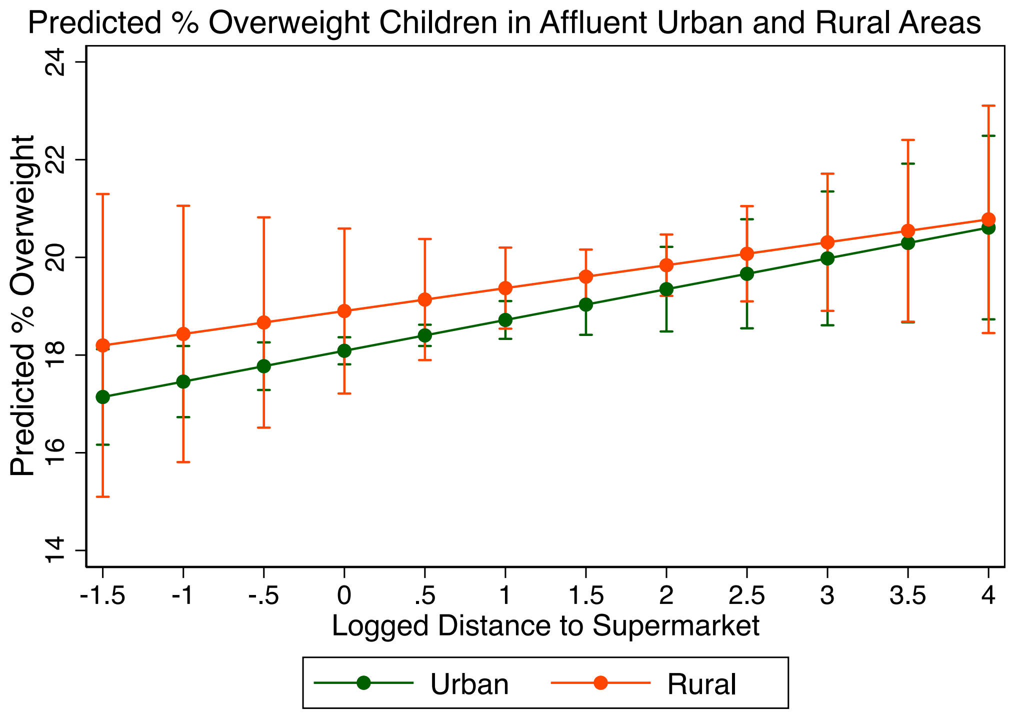

Supplement: Supplementary file 1 [file S1368980023000952sup001.pdf]
